# Longitudinal associations between child, parenting, home and neighbourhood factors and children’s screen time through 4 to 7 years of age

**DOI:** 10.1186/s12889-025-22866-2

**Published:** 2025-05-02

**Authors:** Nicole Hayes, Sonia L. J. White, Donna Berthelsen, Jade Burley, Dylan Cliff

**Affiliations:** 1 Australian Research Council Centre of Excellence for the Digital Child, Brisbane, QLD 4059 Australia; 2 School of Education, Faculty of Creative Industries, Education and Social Justice, Queensland University of Technology, Level 4, E Block, Victoria Park Road, Kelvin Grove, Brisbane, QLD 4059 Australia; 3 Early Start, School of Education, Faculty of the Arts, Social Sciences and Humanities, University of Wollongong, Keiraville, NSW 2522 Australia

**Keywords:** Early childhood, Screen time, Parenting consistency, Home learning environment

## Abstract

**Background:**

Screen-based devices have become a pervasive feature in the lives of young children. Understanding the ecological influences determining young children’s screen use is imperative for promoting healthy screen habits. While individual studies have examined various factors influencing children’s screen use across the early years, few studies have explored multiple ecological influences simultaneously. This research examines longitudinal associations between children’s home environment, parent management practices and neighbourhood influences on children’s screen time, through 4 to 7 years of age.

**Methods:**

Analyses draw on data from 2485 children in the Infant Cohort of a nationally representative sample of children and families participating in the *Longitudinal Study of Australian Children* (LSAC). Bivariate and multiple linear regression models were developed to assess contributions of child, parenting, home and neighbourhood variables, measured at 4–5 years, in predicting children’s daily screen time at 6–7 years.

**Results:**

Girls had lower screen time than boys at 6–7 years of age. Higher household socio-economic status, child participation in home learning activities and out-of-home extra-curricular activities, parenting consistency, and screen management practices including restricted bedroom access, presence and enforcement of screen rules, and parent-reported ease in managing screen time were also all significant predictors of lower daily screen time at age 6–7 years. Neighbourhood factors including liveability, sense of belonging and area-based socio-economic index were not significant predictors of children’s screen time, after child, parenting and home factors were included in the model.

**Conclusions:**

Providing guidance to families about building healthy screen practices from an early age should focus on parenting consistency and management of screen time, as well as responsiveness to engage children in other activities. Home routines on screen use, when consistently managed, can provide young children with opportunities to learn to self-regulate their own screen use behaviours from an early age and build healthy screen time habits.

## Background

Children’s access to screen-based devices has increased considerably over recent decades and the time spent on these devices is a prominent aspect of contemporary childhoods. In Australia, research has indicated that children are spending up to 30% of their waking time engaged in screen-based activities, such as watching TV and movies, and playing video games [1]. More than 50% of Australian parents have also reported that their child does not spend a day without screen-based technology [2]. Screen time is considered to displace the time available for other important developmental experiences, especially engaging in social interactions and outdoor activities, with many concerns focused on increases in children’s solitary and sedentary behaviours [3]. While screen-based activities can be designed for educational purposes and to stimulate imagination and creativity [4, 5], their pervasive use has raised concerns about the impacts of excessive screen time for children’s development, with studies showing negative impacts on physical, cognitive and psychosocial development [6–8]. Excessive screen use has been linked to increased sedentary behaviour [9], poorer sleep [7], lower executive function [10] and language skills [11], and externalising and internalising behaviour problems [12], underscoring the need to support balanced and developmentally appropriate digital engagement. Early childhood is a critical time in which health behaviours and home routines are established that can persist through childhood and adolescence [13–15]. Setting the foundations for healthy screen use during this early formative developmental period can provide children with opportunities to self-regulate their own screen time with important implications for longer-term development and wellbeing.

The bio-ecological systems model [16, 17] provides an important theoretical framework for understanding screen time behaviour in children by focusing on the social contexts in which children live. The model considers the multiple interconnected systems that surround the child and posits that a child’s behaviour is influenced by factors at the intrapersonal level as well as factors within children’s immediate micro-systems and their wider socio-cultural environment. Children’s early home environment is the primary social context in which young children engage with screen-based devices and parents have an important role as mediators of such activities [18]. The home environment is multifaceted and can be differentiated by relatively stable structural indicators such as family resources and household characteristics which create an environment for screen use behaviours to develop through availability and accessibility of devices, while more proximal factors such as parenting beliefs and practices influence children’s screen use through the provision and regulation of screen-based activities [19]. The broader community and neighbourhood environment in which a family lives can also influence children’s screen use by affording opportunities to support engagement in non-screen-based social interactions and physical activities outside of the home. In the Australian context, research has shown that parental perceptions of favourable neighbourhood features such as walkability and access to parks and a sense of community and neighbourhood social interactions are associated with increased outdoor play and reduced screen time among children [20, 21].

While a range of ecological factors have been found to influence children’s screen use in the early years [22–25], few studies have explored the multiple levels of influence at the intrapersonal, immediate micro-system and wider socio-cultural environment simultaneously to understand their relative contributions to variability in children’s daily screen time. Recent systematic reviews have consistently identified proximal influences, such as screen availability and parental rules about use as key determinants of young children’s screen time [25]. Other correlates include a greater breadth of family and socio-cultural demographics and neighbourhood characteristics [22, 23]. However, findings across studies have been inconsistent, suggesting that some influences depend on interactions with other individual, family and environmental factors, related to sample-specific characteristics. The aim of the current study is to examine the longitudinal associations of early child, parenting, home and neighbourhood influences at 4 and 5 years on children’s later screen time at 6–7 years of age, using a large population-level, representative sample of Australian children. With digital technology now a regular part of young children’s lives, identifying salient influences on screen time can be used to inform evidence-based public health initiatives to support families in creating healthy screen time habits during an important developmental period for young children.

## Method

### Study design

These analyses draw on data from the Longitudinal Study of Australian Children (LSAC), a nationally representative prospective cohort study of children’s development from early childhood to young adulthood. LSAC’s study design and sampling frame has been previously described [26]. Data collection commenced in 2004 and the study used a two-stage cluster sampling design to recruit two cohorts of Australian children– an Infant cohort, aged 6–18 months (*n* = 5107) and a Kindergarten cohort, aged 4–5 years (*n* = 4983). The primary sampling unit is Australian postcodes, stratified by state and location (urban and rural split) of residence. Children were then randomly selected using the Australian Medicare universal healthcare database held by the Health Insurance Commission, in which > 90% of infants and 98% of 4-year-old children are registered. The recruited sample in each of the two cohorts were broadly representative of the Australian population, with the exception of children living in remote areas [27].

Participating families are followed in biennial waves, with data collected through home-based interviews with the primary caregiver, as well as through self-complete questionnaires completed by primary caregivers and teachers, and through direct child assessments. Mail-out questionnaires were also completed by the primary caregiver at a mid-point between the biennial data collections of Waves 3 and 4. The current analyses use data from Wave 3 (4–5 years), Wave 3.5 (5–6 years), and Wave 4 (6–7 years), collected in years 2008, 2009 and 2010, from the Infant Cohort of LSAC.

### Measures

#### Child screen time

Children’s screen time was measured at Wave 4 during the primary caregiver home interview, when children were aged 6–7 years. Six items asked caregivers to report the total number of hours that their child spent: (1) watching television, DVDs/videos (television viewing); (2) using a computer, and (3) playing electronic games, separately for a typical weekday and weekend day. Average daily scores were calculated by summing hours reported for an average weekday (multiplied by five) and average weekend day (multiplied by two) and then dividing by seven to obtain a daily average for each form of screen exposure. Average daily television viewing time, computer use and time playing electronic games were summed to create a total score for daily screen time for each child.

#### Predictors of screen time

Predictors of children’s screen time were selected a-priori based on previous research [22, 23, 25] and are detailed in Table 1. These predictor variables were measured at Wave 3 and Wave 3.5 from the primary caregiver home interview and mail-out questionnaire. Drawing upon an ecological framework that focuses on the interplay of intrapersonal, micro-system and broader socio-cultural factors in determining children’s behaviour and development [16, 17], potential predictor variables were classified into child, parenting, home and neighbourhood factors.

**Table 1:** Potential predictors of children’s screen time

Construct	Measure
**Child Factors (Wave 3, 4–5 yrs)**	
Age	Age in months
Sex	0 = Boy; 1 = Girl
Indigenous background	0 = non-Indigenous; 1 = Indigenous
Home language	0 = English; 1 = non-English
Attention regulation	Average of four items from an adapted version of the Short Temperament Scale for Children [43] indicating the degree of persistence the child displays when completing tasks and activities (e.g., “when this child starts a project such as a puzzle he/she works on it until it is completed even if it takes a long time) rated on a 6-point scale from 1 = almost never to 6 = almost always. Higher scores indicating stronger attention regulation skills. α = 0.79
Emotion regulation	Average of four items from the adapted version of the Short Temperament Scale for Children [43] indicating the degree of negative reactivity the child exhibits (e.g., “when shopping together if I do not buy what this child wants he / she cries and yells.”) rated on a 6-point scale from 1 = almost never to 6 = almost always. Scores were reverse coded, with higher scores to indicating stronger emotion regulation. α = 0.68
**Parenting and Home Environment (Wave 3**,** 4–5 yrs)**	
Parenting consistency	Average of five items from the National Longitudinal Survey of Children and Youth [44] in which parents rate the extent to which they followed through with behavioural consequences for the child (e.g., when you give this child an instruction or make a request to do something, how often do you make sure that he/she does it? ) on a 5-point scale from ‘never’ to ‘always’. Higher scores represent higher parenting consistency. α = 0.68.
Home learning activities	Average of five items indicating frequency of adult-child engagement in learning-related activities in the home (reading, music, art and crafts), rated on a 5-point scale from ‘not in the past week’ to ‘6–7 days’. α = 0.68.
Out-of-home extra-curricular activities	Sum of five items indicating child participation (0 = No, 1 = Yes) in extra-curricular swimming, gymnastics, team sport, music and dance.
Parent mental health	Average of six items from the Kessler K6 screening scale [45] about respondents’ feelings over the past four-week period (e.g., in the past 4 weeks, how often have you felt hopeless? ), rated on a 5-point scale from ‘none of the time to ‘all of the time’. Higher scores represent higher mental health symptoms. α = 0.81.
Single-parent household	0 = Two-parent household; 1 = One-parent household
Number of siblings	Number of siblings living in the household
Household socio-economic position	Derived variable within LSAC that combines parental occupational prestige, parental education level and household income, and weighted according to household composition (e.g., single- or two-parent household) [46]. It has an approximate mean of zero and standard deviation of one. Higher scores represent higher socio-economic position.
*Screen Management Practices (Wave 3.5*,* 5–6 yrs)*	
Bedroom access	Sum of four items indicating access (0 = no, 1 = yes) to TV, DVD player, computer and electronic game console in the child’s bedroom.
Adult involvement when using screens	Average of three items indicating how often an adult watches TV/DVDs with child, helps child with the computer and plays electronic games with child, rated on a 5-point scale, from 1 = never to 5 = always. α = 0.61.
Rules about screen use	Sum of five items asking if there are family rules (0 = no; 1 = yes) about quantity and content of screen use.
Enforcement of screen use rules	Average of two items asking parent how often they make sure the child follows rules about screen use rated on a 5-point scale from 1 = Never to 5 = all the time. α = 0.77.
Ease with screen use management	Average of three items asking parents how easy they find managing their child’s screen use rated on a 4-point scale from 1 = very difficult to 4 = very easy. α = 0.84.
**Neighbourhood Environment (Wave 3**,** 4–5 yrs)**	
Liveability	Average of eight items from AIFS Families, Social Capital and Citizenship Survey [47] measuring neighbourhood environment such as cleanliness, street lighting and parks and playgrounds for children to play in, rated on 4-point scale from ‘strongly disagree’ to ‘strongly agree’. α = 0.66.
Sense of belonging	Average of four items adapted from the AIFS Families, Social Capital and Citizenship Survey [47] measuring identity with the neighbourhood (e.g., you feel a strong sense of identity with your neighbourhood; you are well informed of local affairs) rated on a 4-point scale from ‘strongly disagree’ to ‘strongly agree’. α = 0.68.
Socio-economic index for area (SEIFA)	Derived variables with LSAC computed by the Australian Bureau of Statistics [48] that includes a composite of 31 variables (e.g., income, unemployment, occupation and education).

### Data analytic approach

#### Sample selection and missing data

A total of 2485 primary caregivers completed the Wave 3, 3.5 and 4 home interview and questionnaire which collected information on the variables of interest. Participants in the analytic sample were more likely to have a higher socio-economic position, F(1,5092) = 391.485, *p* <.001; and were less likely to be Indigenous, χ^2^(1, 5107) = 84.06, *p* <.001, or have a language other than English at home, χ^2^(1, 5104) = 77.335, *p* <.001, compared to the original sample recruited for Wave 1 data collection.

#### Statistical Analysis

All variables listed in Table 1 were considered as potential predictors. Descriptive statistics were reported as means and standard deviations for continuous variables and frequencies for dichotomous variables. Bivariate correlations were used to explore associations between continuous variables used in the analyses. Linear regression was conducted to explore potential relationships between the child, parenting, home and neighbourhood factors and children’s total daily screen time as the outcome measure. All potential predictors that reached significance in the bivariate regression models (at *p* <.05) were included in a multiple linear regression. All relevant predictor variables were entered simultaneously, allowing for an evaluation of each predictor’s unique contribution to explaining children’s total daily screen time. Analyses were performed using IBM SPSS Statistics (Version 29, IBM, Armonk, NY), and *p*-values were two-tailed, with *p* <.05 considered statistically significant. Regarding model assumptions, the histogram of the residuals appeared approximately bell-shaped, and the normal P-P plot showed a close fit to the diagonal line, suggesting that the residuals were approximately normally distributed. The scatterplot of standardised residuals showed that the data met the assumptions of homogeneity of variance and linearity. Multicollinearity was not a concern, with all VIFs below 2.

## Results

Descriptive statistics for the variables used in the analyses are presented in Table 2. Children’s mean total screen time at 6–7 years of age was 2.57 h (SD = 1.41) per day. Television viewing made up most of children’s screen time, with a mean of 1.77 h (SD = 0.99) per day. Children spent a similar amount of time using a computer and playing electronic games with a mean of 0.42 h (SD = 0.48) and 0.39 h (SD = 0.49) per day, respectively. Bivariate correlations between continuous variables are presented in Table 3. Correlations were in the expected directions and almost all were significant (*p* <.05).

**Table 2:** Sample characteristics and descriptive statistics

Variables of Interest	M (SD) / % (*n*)
**Child Factors**	
Age in months (at Wave 4 data collection)	81.71 (3.44), 73–92
Girls	47.8% (1187)
Non-English home language	6.8% (170)
Indigenous background	1.8% (44)
Attention regulation	3.91 (0.85), 1–6
Emotion regulation	4.46 (0.85), 1–6
**Parenting and Home Environment**	
Parenting consistency	4.21 (0.60), 1.80-5.00
Home learning activities	1.66 (0.55), 0–3
Extra-curricular activities	1.05 (0.90), 0–5
Parent mental health	8.95 (2.99), 6–30
Single-parent household	7.5% (186)
Number of siblings in household	1.43 (0.89), 0–7
Household socio-economic position	0.21 (0.96), -3.81-2.99
*Screen Management Practices*	
Bedroom access	0.18 (0.48), 0–3
Adult involvement	3.08 (0.78), 1–5
Rules about screen use	4.27 (1.08), 0–5
Enforcement of screen use rules	4.47 (0.57), 1–5
Ease with screen use management	3.47 (0.53), 1–4
**Neighbourhood Environment**	
Liveability	3.05 (0.53), 1–4
Sense of belonging	2.91 (0.51), 1–4
SEIFA	1019.73 (74.49), 790–1210
**Screen Time (hours per day)**	
Total screen time	2.57 (1.41), 0-11.44
TV viewing	1.77 (0.99), 0-9.36
Computer use	0.42 (0.48), 0-5.14
Playing electronic games	0.39 (0.49), 0–4.00

Note. SEIFA = Socio-economic index for area; *n* = 2485

**Table 3:** Bivariate correlations between continuous variables of interest

	1	2	3	4	5	6	7	8	9	10	11	12	13	14	15	16	17
1 Child age	-																
2 Attention regulation	0.03	-															
3 Emotion regulation	0.01	0.24**	-														
4 Parenting consistency	0.01	0.18**	0.36**	-													
5 Home learning activities	− 0.04	0.14**	0.13**	0.16**	-												
6 Extra-curricular activities	0.07**	0.07**	0.08**	0.06**	0.12**	-											
7 Mental health	0.02	− 0.09**	− 0.22**	− 0.19**	− 0.04*	− 0.08**	-										
8 Number of siblings	0.03	0.05*	− 0.02	− 0.00	− 0.09**	− 0.10**	− 0.01	-									
9 Household socio-economic position	0.00	0.10**	0.09**	0.14**	0.13**	0.23**	− 0.08**	− 0.02	-								
10 Bedroom access	0.04*	− 0.01	− 0.09**	− 0.12**	− 0.07**	− 0.06**	0.05*	0.01	− 0.21**	-							
11 Adult involvement	− 0.02	0.05*	− 0.00	0.00	0.16**	− 0.04*	− 0.00	− 0.09**	− 0.17**	0.03	-						
12 Rules about screen use	0.07**	0.11**	0.09**	0.10**	0.09**	0.08**	− 0.03	0.05**	0.11**	− 0.04*	0.01	-					
13 Enforcement of screen use rules	0.01	0.09**	0.14**	0.23**	0.16**	0.04	− 0.05*	− 0.03	0.02	− 0.02	0.17**	0.20**	-				
14 Ease with screen use management	0.02	0.14**	0.27**	0.24**	0.09**	0.06**	− 0.18**	0.06**	− 0.05**	− 0.02	0.11**	0.07**	0.35**	-			
15 Neighbourhood liveability	0.01	0.02	0.05**	0.04	0.08**	0.09**	− 0.08**	− 0.06**	0.19**	− 0.03	0.01	0.05*	− 0.02	0.02	-		
16 Neighbourhood belonging	− 0.00	0.08**	0.09**	0.09**	0.14**	0.04*	− 0.14**	0.04*	0.13**	− 0.04*	− 0.01	0.07**	− 0.07**	0.10**	0.28**	-	
17 SEIFA	− 0.01	0.04	0.04*	0.02*	0.08**	0.22**	− 0.06**	− 0.07**	0.39**	− 0.15**	− 0.08**	0.08**	0.00	− 0.06**	0.35**	0.08**	-
18 Total screen time	0.02	− 0.09**	− 0.15**	− 0.17**	− 0.14**	− 0.16**	0.09**	− 0.03	− 0.23**	0.18**	0.01	− 0.12**	− 0.16**	− 0.16**	− 0.02	− 0.06**	− 0.13**

Note. *p <.05, **p <.01; SEIFA = Socio-economic index for area; *n* = 2485

### Predictors of screen time

Table 4 presents the results of the bivariate linear regression analyses. There were no significant associations between young children’s daily screen time at 6–7 years and the following variables: child age, Indigenous background, number of siblings in the home, adult involvement when using screens and neighbourhood liveability. These variables were not included in the final multiple linear regression model.

**Table 4:** Bivariate regression model for the predictors of children’s screen time at 6–7 years of age

		B	SE		β	t	*p*	95% CI
**Child Factors**								
Age in months		0.006	0.008		0.015	0.763	0.446	-0.010–0.022
Girls		-0.312	0.056		-0.113	-5.660	< 0.001	-0.429 - -0.208
Non-English home language		0.257	0.112		0.046	2.298	0.022	0.038–0.477
Indigenous background		0.355	0.214		0.033	1.658	0.097	-0.065–0.776
Attention regulation		-0.139	0.033		-0.084	-4.219	< 0.001	-0.204 - -0.075
Emotion regulation		-0.243	0.033		-0.147	-7.397	< 0.001	-0.307 - -0.178
**Parenting and Home Environment**								
Parenting consistency		-0.406	0.046		-0.173	-8.756	< 0.001	-0.497 - -0.315
Home learning activities		-0.446	0.051		-0.174	-8.813	<0.001	-0.546 - -0.347
Out-of-home extra-curricular activities		-0.251	0.031		-0.159	-8.042	< 0.001	-0.313 - -0.190
Parent mental health		0.043	0.009		0.092	4.598	< 0.001	0.025–0.062
Single-parent household		-0.251	0.107		-0.047	-2.338	0.019	-0.462 - -0.040
Number of siblings		-0.054	0.032		-0.034	-1.689	0.091	-0.116–0.009
Household socio-economic position		-0.333	0.029		-0.227	-11.620	< 0.001	-0.389 - -0.277
*Screen Management Practices*								
Bedroom access		0.536	0.058		0.183	9.269	< 0.001	0.423–0.650
Adult involvement when using screens		0.025	0.037		0.013	0.671	0.503	-0.047–0.097
Rules about screen use		-0.161	0.026		-0.123	-6.193	< 0.001	-0.212 - -0.110
Enforcement of screen use rules		-0.394	0.049		-0.159	-8.045	< 0.001	-0.490 - -0.298
Ease with screen use management		-0.445	0.055		-0.160	-8.075	< 0.001	-0.553 - -0.337
**Neighbourhood Environment**								
Liveability		-0.051	0.054		-0.019	-0.955	0.340	-0.156–0.054
Belonging		-0.158	0.056		-0.057	-2.836	0.005	-0.267 - -0.049
SEIFA		-0.003	0.000		-0.133	-6.703	< 0.001	-0.003 - -0.002

Note. SEIFA = Socio-economic index for area; *n* = 2485

Table 5 presents the results of the multiple linear regression analysis. The multiple linear regression model was significant, F(16, 2484) = 26.121, *p* <.001; adjusted R^2^ = 0.139. In this analysis, significant predictors of children’s daily screen time included child sex, child participation in home learning activities and out-of-home extra-curricular activities, parenting consistency, household socio-economic position, and screen management practices including bedroom access, screen use rules, enforcement of screen use rules and parent-reported ease of management of screen use.

**Table 5:** Multiple variable regression model for the predictors of children’s screen time at 6–7 years of age

	B	SE	β	t	*p*	95 CI
**Child Factors**						
Girls	-0.255	0.054	-0.090	-4.699	< 0.001	-0.361 - -0.148
Non-English home language	0.124	0.105	0.022	1.181	0.238	-0.082–0.330
Attention regulation	0.005	0.032	0.003	0.156	0.876	-0.058–0.069
Emotion regulation	-0.058	0.035	-0.035	-1.684	0.092	-0.127–0.010
**Parenting and Home Environment**						
Parenting consistency	-0.138	0.049	-0.059	-2.795	0.005	-0.235 - -0.041
Home learning activities	-0.192	0.051	-0.075	-3.774	< 0.001	-0.291 - -0.092
Out-of-home extra-curricular activities	-0.100	0.031	-0.063	-3.196	0.001	-0.162 - -0.039
Parent mental health	0.014	0.009	0.030	1.522	0.128	-0.004–0.032
Single-parent household	0.136	0.104	0.025	1.309	0.191	-0.068–0.341
Household socio-economic position	-0.223	0.032	-0.152	-7.009	< 0.001	-0.285 - -0.160
*Screen Management Practices*						
Bedroom access	0.346	0.057	0.118	6.102	< 0.001	0.235–0.457
Rules about screen use	-0.076	0.025	-0.058	-3.011	0.003	-0.125 - -0.026
Enforcement of screen use rules	-0.189	0.051	-0.077	-3.707	< 0.001	-0.290 - -0.089
Ease with screen use management	0.250	0.059	-0.090	4.263	< 0.001	0.135–0.366
**Neighbourhood Environment**						
Belonging	0.027	0.054	0.010	0.495	0.620	-0.078–0.131
SEIFA	-0.001	0.000	-0.034	-1.637	0.102	-0.001–0.000

Note. SEIFA = Socio-economic index for area; *n* = 2485

Girls were more likely to have lower total screen time than boys (β = -0.09, *p* <.001). More frequent participation in home learning activities and out-of-home extra-curricular activities were both associated with lower screen time (β = -0.075, *p* <.001 and β = -0.063, *p* =.001, respectively). Higher parenting consistency was associated with lower screen time (β = -0.059, *p* =.005). A higher household socio-economic position was also associated with lower screen time (β = -0.152, *p* <.001). Access to more screen-based devices in the bedroom was associated with higher total screen time (β = 0.118, *p* <.001), while having more rules about screen use (β = -0.058, *p* <.05) and more frequent enforcement of screen use rules (β = -0.077, *p* <.001) were associated with lower total daily screen time. Parent-reported ease with child screen use management was associated with lower total daily screen use (β = -0.09, *p* <.001).

There were no significant associations between home language, child attention and emotion regulation, number of parents in the household, primary caregiver mental health, neighbourhood sense of belonging and the socio-economic index for the area (SEIFA) with children’s daily screen time (all *p* >.05), after controlling for all other predictor variables.

## Discussion

The current study explored child, parenting, home and neighbourhood predictors of young children’s screen time in a large national sample of Australian families. At the time of data collection (2010), 6–7 year-old children had an average of 2.6 h per day of screen time. Girls were found to have lower screen time than boys. Children in families who had higher socio-economic status also had lower daily levels of screen use, with SES emerging as the most significant predictor in the model. Investigation of home and parenting management practices related to screen time identified that lower screen time was associated with a more consistent parenting style (e.g., following through on instructions given to child and ensuring compliance), higher engagement with children in home learning activities and higher child participation in out-of-home activities. Parents of children with lower levels of screen time also reported having more rules about screen time, consistent enforcement of screen time rules, and greater ease in managing screen time, which were some of the strongest predictors of lower screen use. In examining longitudinal associations for a comprehensive set of ecological factors simultaneously on children’s screen time, the findings from the current study have important implications for public health initiatives and intervention efforts that aim to support healthy screen use habits in young children. While the overall model explained a modest amount of variance in children’s screen time, the findings identify several key modifiable factors that can inform targeted intervention efforts.

While data in the current study were collected in 2010, the average level of daily screen time usage for Australian children at this time is in line with recent international findings. Data collected in early 2020 from a nationally representative survey of parents and children in the United States [28] reported that average daily screen time for children aged 8 years and younger was 2 h 24 min and this usage was also primarily television and video viewing. The researchers also noted that this amount of screen time usage had remained relatively stable, for this age group, over the nine years in which this survey in the United States had been conducted. However, it is important to acknowledge that the proliferation of smartphones and mobile devices, as well as the global COVID-19 pandemic, has likely altered screen time dynamics, especially in relation to hand-held devices and increases in excessive screen time concerns among children [29, 30]. The findings from the current study provide a foundational understanding of screen time behaviours that can be used to compare with more recent data to explore shifts in usage patterns, parenting challenges and child development in response to evolving digital environments.

In the current study, the finding that boys had higher overall screen time than girls may reflect the type of screen time measured in the current study that included TV viewing, electronic game playing and computer use. Previous systematic reviews of correlates of screen use during early childhood [22, 23, 25] have consistently reported no association with child sex, although most studies included in these reviews measured traditional TV viewing only. Studies that distinguish between different types of screen-based activities have found that, while television viewing is similar across sex, boys tend to spend more time playing video games than girls and that these differences increase with age [31, 32].

In the bivariate analyses, poorer attention and emotion regulation were associated with higher screen time; however, this association did not remain significant in the adjusted model. This may be related to the developmental stage of the sample and the role of adults in enabling access to screen-based devices for this age group. Research with young children (aged 5 and under) has found that parental monitoring and limit setting are associated with children’s screen time [33]. As children grow older, individual self-regulatory ability may play a more influential role in predicting screen time, as supervision decreases and access to devices increases. This underscores the importance of further detailed longitudinal analyses in this area to examine if children’s increasing capacities for self-regulation affect the level of parental involvement and how parents act to monitor screen time across developmental stages.

Parental engagement with their child in home learning activities, as well as child participation in out-of-home extra-curricular activities were associated with lower screen time, aligning with existing research that emphasises the role of non-screen-based activities in promoting healthier screen time behaviours. For example, increased access to cognitively stimulating activities in the home, such as shared reading and educational play, and interventions that promotion parent-child interaction through these activities have both been associated with reduced screen use among young children and infants [34, 35] Such interactional opportunities with others, both inside and outside of the home, not only displaces screen time but also provide children with opportunities for physical movement and social engagement.

The findings also identified a significant association between family socio-economic status (SES) and screen time, similar to previous research [28]. SES represents the social, cultural and economic capital available in families and is an important factor that affects the resources parents can invest in their children [36]. Higher SES enables families greater access to alternative non-screen-based resources and activities. As a consequence, children from higher SES backgrounds are more likely to have lower reliance on screen-based entertainment. Notably, SES had one of the strongest effects in the model, reinforcing its central role in shaping children’s screen behaviours and the need for intervention efforts to consider broader socio-economic factors.

Consistent with previous research [25, 37], the current study found parental screen management practices related to restricted bedroom access, and the presence and enforcement of screen use rules were associated with lower time spent in screen-based activities. Crucially, one of the strongest predictors of lower screen time was parent-reported ease in managing their child’s screen use, suggesting that parental self-efficacy in managing screen habits plays an important role. Providing guidance to families around healthy screen habits in the home as well as increasing parental confidence in managing their child’s screen use may be potential strategies for future interventions. Broader parenting behaviours were also significantly associated with children’s screen time in the adjusted models, supporting the importance of consistent routines and practices in the home in facilitating the development of healthy habits in children [38–40].

Measures focused on the neighbourhood environment of families including the area-based socio-economic index (SEIFA), as well as parental perceptions of belonging and liveability of the wider neighbourhood environment were not associated with children’s screen time in the adjusted models, suggesting that more proximal factors within the family microsystem and home environment may likely offer the most effective targets for intervention to reduce screen time of young children, and may have more relevance and impact on older children and adolescents. The measures used in the current study, however, did not examine aspects of neighbourhood safety and crime, which may influence parental decisions regarding the provision of screen-based activities inside the home compared to outdoor play [20].

### Strengths and limitations

The current study has several strengths including the use of a large cohort, the inclusion of multiple independent predictor variables, and the exploration of longitudinal relations between these predictor variables and children’s screen time. There are limitations, however, that should be considered when interpreting the findings. Children’s screen time was measured using subjective parent-report, which have been shown to both underestimate and overestimate exposure because of recall bias, social desirability bias or simply not being aware of screen viewing behaviours [41, 42]. The participants in this study were recruited in 2004, with data collection about children’s screen time at 6–7 years of age occurring in 2010. Since then, there has been significant change in the availability and type of screen-based devices and this means we have not captured time spent on more contemporary devices, such as smartphones and tablets. It is also important to note that quantity of screen time represents one aspect of children’s screen use behaviours. Beyond quantity, examining the contextual factors that influence screen time, such as the content (i.e., educational, entertainment), level of engagement (i.e., passive, active), and co-use (i.e., in the presence of peers or parents) will be important for future investigations. It is also possible that the association between the independent predictor variables and children’s screen time may be over-estimated due to common method variance with data across these variables collected from the same informant. Finally, although we used data from a well-defined birth cohort enabling the results to be generalised to a national population, participants from lower socio-economic groups and culturally diverse backgrounds were less likely to have complete data on the variables used in this study. Thus, the results may not generalise to these populations.

## Conclusion

The current study explored child, parenting, home and neighbourhood predictors of young children’s screen time in a large national sample of Australian families. The findings suggest that supporting parents to implement screen management practices in the home, including limiting children’s access to screen-based devices in bedrooms, and having and enforcing rules about children’s screen use, may be potential targets to foster healthy screen time habits in young children. Support in these areas may contribute to increasing parent’s perceived ease in managing children’s screen use, which was also associated with children’s screen time. Additionally, strategies to reduce children’s screen time should include understandings of the importance of engaging children in alternative non-screen-based activities in the home that foster children’s learning in areas in which they have strong interests, as well as for engagement in out-of-home extra-curricular activities and community events. Given the study’s large, nationally representative sample, these findings can inform public health initiatives aimed at promoting balanced screen use in the early years through family-focused interventions that support parents in managing both screen and non-screen-based engagement with their children. Moreover, these results, which focus on screen use via televisions, computers and gaming consoles, provide a foundation for future research to examine the evolving digital landscape, including the growing use of mobile devices and how factors such as parenting practices and home environments continue to shape children’s screen use in the digital age. Later waves of data collection in the LSAC dataset offer opportunities to explore how early screen use influence subsequent digital engagement and development outcomes through to adolescence.

## Data Availability

The dataset analysed for the current study is available from the Australian Data Archive at https://dataverse.ada.edu.au/dataverse/lsac.
